# From Endoderm to Progenitors: An Update on the Early Steps of Thyroid Morphogenesis in the Zebrafish

**DOI:** 10.3389/fendo.2021.664557

**Published:** 2021-06-04

**Authors:** Federica Marelli, Giuditta Rurale, Luca Persani

**Affiliations:** ^1^ Dipartimento di Malattie Endocrine e del Metabolismo, IRCCS Istituto Auxologico Italiano IRCCS, Milan, Italy; ^2^ Dipartimento di Biotecnologie Mediche e Medicina Traslazionale, Università degli Studi di Milano - LITA, Segrate, Italy

**Keywords:** congenital hypothyroidism, thyroid development, endoderm, Notch, sonic hedgehog, fibroblast growth factor, bone morphogenic protein, Wnt/b-catenin

## Abstract

The mechanisms underlying thyroid gland development have a central interest in biology and this review is aimed to provide an update on the recent advancements on the early steps of thyroid differentiation that were obtained in the zebrafish, because this teleost fish revealed to be a suitable organism to study the early developmental stages. Physiologically, the thyroid precursors fate is delineated by the appearance among the endoderm cells of the foregut of a restricted cell population expressing specific transcription factors, including *pax2a*, *nkx2.4b*, and *hhex*. The committed thyroid primordium first appears as a thickening of the pharyngeal floor of the anterior endoderm, that subsequently detaches from the floor and migrates to its final location where it gives rise to the thyroid hormone-producing follicles. At variance with mammalian models, thyroid precursor differentiation in zebrafish occurs early during the developmental process before the dislocation to the eutopic positioning of thyroid follicles. Several pathways have been implicated in these early events and nowadays there is evidence of a complex crosstalk between intrinsic (coming from the endoderm and thyroid precursors) and extrinsic factors (coming from surrounding tissues, as the cardiac mesoderm) whose organization in time and space is probably required for the proper thyroid development. In particular, Notch, Shh, Fgf, Bmp, and Wnt signaling seems to be required for the commitment of endodermal cells to a thyroid fate at specific developmental windows of zebrafish embryo. Here, we summarize the recent findings produced in the various zebrafish experimental models with the aim to define a comprehensive picture of such complicated puzzle.

## Introduction

In all vertebrates, the thyroid gland provides the production of thyroid hormones (TH), thyroxine (T4) and its active metabolite triiodothyronine (T3), which exerts multiple roles during embryonic growth and organ development, and influencing several physiological mechanisms, including neurological development, metabolism, reproduction, thermoregulation, cardiac function, and tissue repair (1).

Following the fundamental steps of embryonic development, the establishment of a complex network intrinsic and extrinsic signals from surrounding tissues is the prerequisite for the acquisition of cell competence and the subsequent organogenesis of the thyroid gland (2).

In mammals, the development of the thyroid gland begins with the evagination of the primordium from the thyroid diverticulum located on the midline of the pharyngeal floor. The undifferentiated precursors then migrate to reach a position deep in the cervical mesenchyme where they will differentiate into hormone-producing follicles (3). In teleost fish, such as zebrafish, the thyroid gland differs from that in other vertebrates: a) the endodermal precursors start to differentiate before the relocalization; b) the thyroid follicles do not form a compact gland but remain loosely dispersed along the pharyngeal midline (4). However, despite differences in size, shape, or anatomical position, the capacity of thyroid follicles to face the physiological demand of thyroid hormone (TH), as a key prerequisite for normal thyroid function, is well conserved across species (1–3, 5, 6).

In humans, the vast majority of cases with congenital hypothyroidism (CH) is due to thyroid dysgenesis (TD), resulting from defects during embryonic thyroid development. Although next-generation sequencing techniques allow the identification of new candidate genes, TD is frequently reported to be sporadic and causative gene variations has been identified in less than 10% of cases (7). This is, in part, due to our still limited knowledge on the morphogenetic events underlying thyroid organogenesis (2, 6).

Especially, while core aspects of thyroid development have been extensively studied in various species, many significant questions are still open: the intrinsic factors responsible for the regional specification of the endoderm, and the role of extrinsic signals that regulate foregut endoderm patterning, remain to be fully clarified. Our current knowledge about these events derives from a patchwork of different observations obtained through variable experimental approaches in diverse species. For this reason, it is difficult to generate a strong hypothesis about the specific role of each signal during thyroid development, as well as their individual contribution to TD pathogenesis (1, 6, 8–16).

In the last decades, the zebrafish embryo has been extensively used as a model system for thyroid development (1, 5, 8, 10, 13, 17), but it is a relative newcomer among other model organisms to study early endoderm formation and induction (18–20). However, the zebrafish embryo offers many advantages that could overcome some limitations of mammalian models. For instance, the cellular dynamics of mammalian thyroid organogenesis are poorly studied due to the inaccessibility and opacity of the fetal tissues. In contrast, the optical clarity and the external fertilization of zebrafish make the embryos accessible to live monitoring and external manipulation. Moreover, the availability of molecular tools for the genetic manipulation of zebrafish allows the generation of several targeted-genetic mutants and transgenic zebrafish lines expressing fluorescent proteins in specific cell types (12). Noteworthy, the high conservation of the morphogenetic events of zebrafish embryogenesis, including thyroid organogenesis, makes zebrafish a valuable model for investigating the very early phases of thyroid development.

In this review, we attempt to summarize both classical and recent findings of thyroid development in the zebrafish embryo, with a particular focus on the early morphogenetic events that regulate endoderm formation and endodermal cells induction into thyroid precursors.

## Key Features of Thyroid Development in Zebrafish

The analysis of zebrafish thyroid tissue during embryogenesis is particularly difficult due to its small size. However, thanks to the optical clarity of zebrafish embryos, the expression of the different thyroid markers was initially studied using whole mount *in situ* hybridization or immunofluorescence on embryos fixed at the desired developmental stage (5, 6, 8, 21). More recently, the generation of transgenic lines that express the fluorescent reporter under the control of a specific thyroid promoter gene (e.g., *thyroglobulin*, *tg*) allows the real-time and *in vivo* analysis of gene expression, cell morphology and thyroid anatomy (12, 14, 22). Moreover, the combination of double reporter lines [e.g., *tg*(*tg:mCherry;myl7:EGFP*)] permits the study of the anatomical relationship between cardiovascular remodeling and thyroid development (22). Very recently, Haerlingen et al. (16) performed an accurate analysis of the expression of the thyroid transcription factors (TTFs) by performing a cell counting on entire thyroid primordia isolated from immunostained zebrafish embryos and reporter lines (16).

These recent works describe that thyroid organogenesis begins around 20 hours post fertilization (hpf) with the expression of *pax2a* at the level of the foregut endoderm. Around 24 hpf, a monolayer of committed endodermal cells expressing the TTFs *nkx2.4b* and *hhex*, also known as thyroid anlage, are detectable at the level of the pharyngeal floor of the primitive pharynx, close to the apical pole of the primitive heart tube (8, 12, 21–23). The expression of *pax2a* in a relatively large domain of foregut endoderm before the expression of *nkx2.4b* suggests that thyroid specification occurs in two steps: endodermal specification of thyroid precursors expressing *pax2a*, and commitment of precursors to the thyroid fate upon *nkx2.4b* activation (16). Around 28-30 hpf, the thyroid anlage also starts to express *pax8* and *foxe1*, but the functional roles of these TTFs for zebrafish thyroid cell differentiation remain unclear. Around 36-40 hpf, the thyroid anlage expands forming the so-called thyroid placode, a multilayer of thyroid precursors protruding into the underlying mesenchyme and budding from the pharyngeal epithelium in a rostro-ventral direction and starts to express the differentiated thyroid marker *tg*. The first follicular cell (TFC) is formed by 55 hpf and express all of the functional genes like thyroperoxidase (*tpo*), Na/I symporter (*slc5a5*), and thyroid stimulating hormone receptor (*tshr*) required for the establishment of competence for TH synthesis. During the later stages of embryonic development and larval transition (55-120 hpf), the thyroid tissue proliferates doubling the number of TFCs and migrates posteriorly forming distinct follicular units scattered along the pharyngeal midline, moving close and along the ventral aorta (12, 14, 16, 22).

Loss of function experiments indicate that zebrafish TTFs have similar roles to their mammalian homologues *NKX2-1*, *PAX8*, and *HHEX*, respectively. Zebrafish models with defective expression of zebrafish TTFs exhibit defects in early thyroid morphogenesis resulting in severe thyroid hypoplasia or athyreosis. The morpholino-mediated knockdown of *nkx2.4b*, *pax2a*, or *hhex* cause a failure of thyroid primordium specification, whereas the abrogation of *hhex* results in a reduced formation of follicles (4, 8, 13, 24). More recently, athyreosis or thyroid hypoplasia are observed in about the 50% of somatic mutants of *nkx2.4b* or *pax2a* generated using the CRISPR/Cas9 technology (14).

## Early Events of Zebrafish Thyroid Development

As a general concept, the complete development of endoderm-derived organs is a multistep process: 1) induction of endoderm, 2) endoderm reorganization into specific regions, 3) start of organogenesis, with differentiation of endoderm precursors. Notably, the differentiation of the endoderm-derived organs, such as thyroid, liver, and pancreas, in the zebrafish embryo starts within the endodermal tissue (8, 25, 26).

Although the main morphogenetic events of thyroid development have been outlined in various model organisms, the precise contribution and interplay of the different molecular mechanisms regulating endodermal cell fate and tissue dynamics processes accounting for the early steps of thyroid development remains to be fully dissected. Moreover, it has been demonstrated that for several organs, including thyroid, the number of precursors that are initially committed from the endodermal layer will determine the final size of the organ (27–29).

Therefore, the understanding of the origin and nature of signals that directly or indirectly drive the very early phases of thyroid morphogenesis is particularly important in thyroid research.

### Endoderm Formation and Cell-Fate Decision

The repertoire of structures derived from zebrafish endoderm is similar to those of mammals, with the exception of the intestinal tube instead of stomach and duodenum, and the swim bladder rather than lungs (30–32).

**Figure 1:**
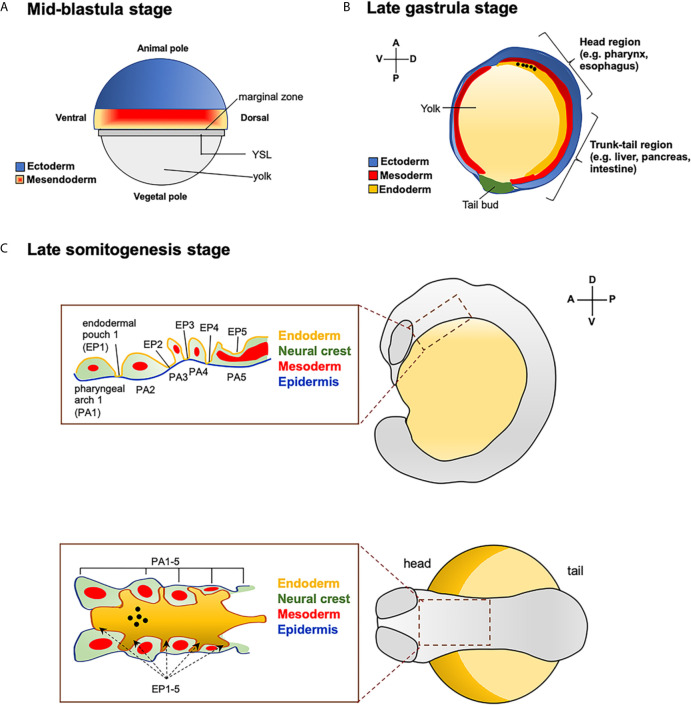
Fate map of zebrafish endoderm. **(A)** Representation of zebrafish blastula in surface view. Position of ectoderm (in blue) and mesendoderm (mesoderm + endoderm pluripotent cells, in red and orange mash-up) at the mid-blastula stage. YLS: yolk syncytial layer. **(B)** Zebrafish embryo at late gastrula stage. Antero-posterior and dorso-ventral movements of the three germ-layers. The presumptive location of endodermal cells that will give rise to the head (e.g., pharynx, esophagus) and trunk-tail (e.g., liver, pancreas and intestine) organs are also reported. The black dots indicate the position of the endodermal cell that contribute to the pharynx, as a site of origin of the thyroid anlage. **(C)** Close up of the pharyngeal endodermal region in embryos at the late somitogenesis (in lateral and dorsal view). Pharyngeal endoderm is surrounded by mesoderm, and neural crest and epidermis (ectoderm). The black dots indicate the position of the future thyroid anlage that will originate from the pharyngeal floor.

To study the induction of thyroid organogenesis, it is important to know “how”, “where”, and “when” the differentiation of endoderm begins. In zebrafish, the establishment of the three primary germ layers occurs between the blastula and the gastrula stages (2.5-10 hpf) of embryonic development. The future ectoderm originates from cells located in the animal pole of early blastula, whereas cells of the marginal region are bipotential for mesodermal and endodermal fates, forming the so-called mesendodermal layer (**Figure 1A**).

A restricted fate of marginal cells is acquired soon after the onset of gastrulation (33). In fact, when marginal cells from gastrulae at 50%-epiboly stage (5.3 hpf) are transplanted into animal blastomeres, they predominantly differentiate into endodermal cells. In contrast, transplanted cells from blastulae at 40% epiboly stage (5 hpf) mostly contribute to neuroectoderm formation (34, 35).

During gastrulation, involution and convergent-extension movements drive the migration of the diverse cell populations into their final locations. At early gastrulation (5.7-6 hpf), both mesodermal and endodermal cells lying the marginal zone start to involute, where the endodermal cells rapidly disperse throughout a “random walk” mechanism. At mid-gastrula stage (8-9 hpf), the endodermal cells converge to form a distinctive flattened morphology and start to express typical endodermal makers (18, 36). At the end of gastrulation (10 hpf), the endodermal cells extend along the antero-posterior axes and differentiate into the various precursor cells that will give rise to the endoderm-derived organs. The endodermal cells located in the dorsal-anterior direction will contribute to the head organs, like pharynx (site of origin of thyroid anlage) and esophagus, while the most ventral-posterior located endoderm will contribute to organs of trunk-tail region, such as liver, pancreas and intestine (**Figure 1B**) (35, 37, 38). At the end of somitogenesis (14-16 hpf) the dorsal-ventral and anterior-posterior axis are completely formed, and both anterior and posterior endodermal cells start to differentiate into their various organ progenitors.

At the level of the pharyngeal region, the endoderm is surrounded by mesoderm, neural crest and epidermis (ectoderm). Five endodermal pouches separate the pharyngeal arches, whose development involves tissues from all three germ layers (**Figure 1C**) (39, 40).

### Critical Aspects of Endoderm Formation

All of the events that shape the developing embryos are regulated by key signaling molecules, also termed morphogens (41). Any pathway relevant to endoderm induction and specification of the pharyngeal endoderm could play a role in thyroid specification. The study of earlier steps of thyroid organogenesis cannot be done in murine models, since mouse embryos with targeted inactivation of genes required for endoderm patterning die in stages prior the definition of the thyroid anlage (42).

Therefore, our knowledge of the morphogenetic events underlie endoderm formation is mostly derived from studies in *Xenopus* and zebrafish (18, 43). Zebrafish has the advantage to combine features of both mammalian and amphibian development. In particular, while mammalian development largely depends on the crosstalk of embryonic and extraembryonic tissues, the induction of early morphogenetic events in amphibians is controlled by maternal factors (33, 44).

In zebrafish, the induction and patterning of the three germ layers rely on signals derived from extraembryonic tissues (yolk and yolk syncytial layer, YLS) and maternal factors initially deposited within the oocyte (41, 45–47). For instance, Nodal/TGFβ ligands, morphogens with critical roles in mesoderm and endoderm specification, are initially expressed under maternal control and then secreted by the extraembryonic YSL to pattern the embryonic blastoderm (48).

The presence of maternal factors during the first hours of development appears to contribute to the survival of embryos wherein their corresponding zygotic genes are inactivated, permitting the analysis of thyroid morphogenesis in later stages (49, 50).

Additionally, before the development of a functional cardiovascular system (around 24 hpf), the zebrafish embryos receive adequate oxygen supply by simple diffusion and can survive for days without blood circulation or a functional heart. In fact, cardiovascular defects are usually tolerated, and the embryos remain viable and develop relatively normally (51, 52). In light of the close relationship between thyroid and heart development, this quality is of particular importance for the study of genes that affect both endoderm and mesoderm development (8, 22).

## Morphogens Involved in Endoderm Induction and Thyroid Specification

### The Nodal Pathway

The generation of several zebrafish models with targeted genetic inactivation or overexpression facilitates the study of the various morphogenetic pathways involved in endoderm development.

At the top of these mechanisms lies Nodal signaling, one of the major contributor of mesoderm and endoderm cell commitment from the bipotential mesendodermal layer, which also plays a role in defining antero-posterior and left-right axes, and neural patterning (53). Nodal signaling is first described in mice with the identification of the activin-like members of the transforming growth factor β (TGF-β) family (54). In zebrafish, cyclops (cyc) and squint (sqt), two nodal-related ligands are necessary to initiate endoderm specification (49). Cyc and sqt bind the *TARAM-A* and the one-eyed pinhead (oep) receptors inducing a signalling cascade mediated by Smad proteins, which culminates with the expression of GATA-binding protein 5 (gata5), and MIX-like (bon, mezzo) transcription factors (55–57). Gata5, bon, and mezzo act in parallel, and in a partially redundant manner, to induce the expression of the transcription factor sox32 (formerly called casanova or cas), which is essential for the transcription of other endoderm-related transcription factors, like sox17 and foxA2 (18, 55).

Thanks to the stability and diffusion of its ligands, Nodal signaling is able to act in a long-range and in a dose-dependent manner. For instance, endoderm specification requires higher levels of Nodal signal than mesoderm (58). Moreover, mechanisms involving the Nodal antagonist *lefty* and the proteolytic inactivation of its ligands finely regulate the spatiotemporal activity of Nodal signaling (53).

Significant knowledge on the role of Nodal pathway during the early steps of thyroid development has been described in the zebrafish model by Elsalini and Rohr (59). The *oep^tz57^* mutant embryos display a complete failure mesendodermal cells commitment that results in the loss of endoderm specification during gastrulation, and absence of thyroid primordium in later stages (59, 60).

Moreover, the disrupted expression of cyclops in *cyc^m294^* mutant leads to an incomplete development of pharyngeal endoderm and reduced number of functional thyroid follicles, whereas the deletion of both *cyc* and *hhex* genes in *cyc^b216^* mutants results in a complete absence of thyroid primordium and no T4-positive follicles in later stages (24, 61). These data suggest that the number of cells initially committed to a thyroid fate restricts the final size, and that a proper thyroid development requires both endoderm specification and expression of early TTFs (24, 62).

Also, the genetic inactivation of the downstream effectors of Nodal signaling *bon*, *gata5* and *sox32* perturbs endoderm organization, specifically leading to an absent or reduced expression of *sox17* and *foxA2*, which prevents the specification of thyroid primordium (24, 53, 57, 63, 64).

However, when the inhibition of Nodal/TGFβ signaling is temporally restricted treating the embryos with chemicals from 50%-epiboly to early somitogenesis, the thyroid specification is only modestly affected despite a marked alteration of anterior endoderm (9). In this developmental window, the development of endodermal progenitors with thyroid commitment appeared to be mostly independent of Nodal/TGFβ signaling. Interestingly, the administration of inhibitors at high dosage results in the formation of ectopic clusters of *nkx2.4b*-positive cells in posterior pharyngeal region, suggesting a possible posteriorizing activity of Nodal signaling during the antero-posterior patterning of the endoderm (9).

### Notch Signaling

In general, the endoderm of zebrafish gastrula is characterized by a “salt-and-pepper” distribution along the marginal zone of the two endodermal markers *sox17* and *foxA2*, which are under the control of the Nodal transcription factors *bon*, *gata5*, and *mezzo* (65). Since only a portion of marginal cells expressing the Nodal genes become endoderm, it seems reasonable that additional genetic programs are implicated in the endoderm-fate restriction of marginal cells (66). Several studies described the key role of the Notch pathway during endoderm development. It has been demonstrated that the Notch signal acts restricting the commitment of endodermal cells through the lateral inhibition of neighboring cells, preventing them to undergo the same fate (67–70).

In vertebrates, there are five Notch ligands, three δ-like (DLL1, DLL3, DLL4) and two Jagged (JAG1 and JAG2), and four Notch receptors (NOTCH1–NOTCH4), with mostly non redundant functions. After the interaction with the ligand, the C-terminal region of Notch receptor, called Notch Intracellular domain (NICD), is released into the cytoplasm and translocates into the nucleus where it induces the expression of the transcriptional repressors belonging to the Hairy and Enhancer of Split (*Hes*) family (70).

In zebrafish, the importance of Notch during embryonic growth and specification of several organs and tissues has been extensively reported. In particular, Notch signaling appears fundamental for the differentiation of primary neuron and oligodendrocyte, the decision between hypochord and notochord cell fates, and the determination of pancreas identity (71–76).

Concerning early endoderm induction, it has been shown that the overexpression of Notch signaling is associated with a reduced number of endodermal cells expressing *sox17* and *foxA2* in zebrafish gastrula. In contrast, the overexpression of *bon*, which acts upstream of *sox17* and *foxA2*, results in a diminished activation of Notch signal. Moreover, *bon*, *gata5*, and *mezzo* are expressed in the same region as *deltaC*, *deltaD*, and *notch1* in the marginal zone (orthologues of mammalian DLL1, DLL3, and NOTCH1, respectively), and *oep* morphants display no expression of Notch receptors (66). Altogether these findings indicate that Notch pathway is directly dependent upon Nodal activity, and that the activation of Notch signaling during early embryonic growth restricts the number of endodermal cells, likely pushing the bipotential mesendodermal cells to become mesoderm (66).

Regarding thyroid development, our research group reported that the hyperactivation or the inactivation of the Notch signaling from about 20 hpf, just before the onset of thyroid primordium, is associated with the reduction or increment of the number of thyroid precursors, respectively (77). The overexpression of NICD in conditional activated zebrafish line abolishes the expression of *nkx2.4b*, together with a significant reduction of mature thyroid follicles. Conversely, the chemical or genetic inhibition of Notch signaling (DAPT or *Mib^ta52b^* mutant) result in an increased expression of *nkx2.4b* and number of *tg*-positive cells at later stages. Given that the constitutive lack of Notch signaling (*Mib* mutants) or its time-controlled inhibition with DAPT at 20 hpf show similar thyroid phenotypes, we assumed that Notch signaling acts just before the endodermal specification of thyroid precursors.

Additionally, we demonstrated the Notch ligands *jag1a* and *jag1b* (homologous of the human *JAG1*) are detectable in the same territories of *tg* but with distinctive roles during thyroid development. *Jag1b* appears to be important to define the number of thyroid precursors that differentiates from pharyngeal endoderm, whereas *jag1a* acts in later stages regulating the renewal and displacement of follicles along the pharyngeal tube (77).

In the light of these findings, Notch signaling appears to act at multiple levels during thyroid development: controlling the differentiation of the primitive endoderm, restricting the number of endodermal cells committed to a thyroid fate, and regulating the expansion and migration of the fully functional thyroid follicles. However, given the roles of Notch in mesoderm induction and differentiation of mesoderm-derived structures, it is also conceivable that thyroid development is influenced by permissive factors from the surrounding tissues (e.g., lateral plate mesoderm) (15, 22, 77).

Haerlingen et al. (9) recently performed an extensive screening of thyroid defects in zebrafish embryos obtained after time-restricted administration of several chemical inhibitors of different morphogenic pathway, including the Notch signaling. The authors described that the treatment with two new Notch inhibitors has no major effects on thyroid development (9). The reasons of conflicting findings to those previously discussed are currently unknown, but differences in experimental condition or compounds activity in the different studies should be also taken into account. However, it is also possible that cardiac and pharyngeal vessels abnormalities observed in DAPT-treated embryos and *Mib* mutants account for the onset of the most severe thyroid phenotype. This view is also supported by the existence of a common pathway regulating thyroid and heart development (22). Nevertheless, the involvement of Jag1-Notch signaling in thyroid organogenesis is also sustained by the enrichment in *JAG1* pathogenic variants among patients with CH, and in particular in syndromic cases with thyroid dysgenesis and associated cardiac defects, as well as by the occurrence of thyroid dysfunction among young patients with Alagille syndrome (13, 77, 78).

### Sonic Hedgehog Pathway

In vertebrates, Sonic Hedgehog (SHH) is known to be an important regulator of embryogenesis, during left-right determination of body axis and organogenesis, and it is also implicated in the development of the pharyngeal apparatus (79–81).

The activation of SHH signaling starts with the binding of SHH to the transmembrane receptor PTCH1 that releases its inhibitory effect on the neighbor receptor SMO. As a result, SMO binds SUFU into the cytosol, negatively regulating its binding to the GLI transcription factors. Once released, GLI translocates into the nucleus and interacts with specific GLI-responsive elements located into the promoter region of target genes, thus activating or repressing their expression (82). Genes of the SHH signaling are expressed during the early developmental stages of zebrafish embryonic development (8). Targeted inactivation of *shh* results in abnormal thyroid migration with absent bifurcation and mislocalization of follicles, likely due to the asymmetrical development of the pharyngeal arches of *shh* mutants (23, 24). Similarly, Haerlingen et al. (9) described that chemical perturbation of SHH pathway with cyclopamine only has minor effects during endoderm formation and specification of thyroid anlage but appears to induce thyroid abnormalities in later stages, likely due to impaired cardiovascular development (9). Moreover, since migration of follicles follows the anatomy of the dorsal aorta, SHH signaling seems to be important for the definition of the foregut territories that sustains thyroid development (6).

However, we recently described a novel implication of SHH signaling in zebrafish thyroid specification (83, 84). The genetic screening in cohorts of patients with syndromic or isolated CH have associated mutations in *GLIS3*, a downstream effector of SHH, and defects in thyroid development (7, 85, 86). In zebrafish, *glis3* is expressed in the pharyngeal endoderm at 22-24 hpf, and knockdown embryos for *glis3* present a reduced number of thyroid precursors expressing *nkx2.4b* and *pax2a*, and low T4-producing follicles at the larval stage. Conversely, the overexpression *glis3* mRNA results in an enlargement of thyroid precursor population and increased number of mature follicles. Interestingly, cyclopamine abolishes the expression of *glis3*, but the exogenous expression *glis3* is able to rescue the thyroid phenotype of cyclopamine treated embryos. Since, both endoderm and cardiovascular development appear unaffected in *glis3* knockdown embryos, these findings suggest a possible direct role of glis3, belonging the SHH pathway, in regulating the amount of endodermal precursors that will differentiate into thyroid cells (83). A similar mechanism for *Glis3* has been described in mice pancreas development, where *Glis3* appears fundamental for β-cell lineage specification and insulin expression (87). Altogether these data highlight the causative role of *GLIS3* biallelic mutations in a complex human phenotype including thyroid dysgenesis together with neonatal type-1 diabetes (NDH syndrome) (85, 86).

Considering the impairment of thyroid specification in *glis3* knockdown embryos and the mislocalization of thyroid follicles observed in previous models, shh signaling seems to be required during early embryonic development for the commitment of thyroid precursors, and later, likely involving different downstream effectors, to control thyroid cell migration along the pharyngeal vessels (6, 9, 83, 88).

### Fibroblast Growth Factors

As already mentioned, the proper development of the endoderm-derived organs needs a source of permissive signals from the surrounding mesoderm. Experiments conducted in primary cell cultures explanted from mouse embryos demonstrated that the inhibition of FGF signal from the cardiac mesoderm prevents the specification of thyroid progenitors, whereas FGF2 induces thyroid primordium specification from the anterior foregut endoderm (89–91). Although FGF signaling is well described in the mesoderm context, only few studies have addressed the role of FGF in endoderm patterning and thyroid development in the zebrafish (35, 56, 92, 93). Wendl and colleagues (2007) reported that, during zebrafish somitogenesis, *fgf8* is detectable in cardiac mesoderm, adjacent the developing anterior foregut endoderm, and that *fgf8 ace^ti282a^* mutants exhibit thyroid dysgenesis (93). Additional findings derived from the observation of embryos with abolished expression of *hand2*, encoding for a bHLH transcription factor expressed in the neural crest mesenchyme of the pharyngeal arches, branchial arteries, and heart tube, and at low levels in the pharyngeal endoderm (94). Zebrafish *hand2^s6^* mutants display defects in thyroid primordium specification in presence of normal endoderm formation. Similarly, in *hand2^c99^* mutants the thyroid primordium is absent, concurrent with defects on pharynx, heart, and fin development (93). Since the endoderm-derived thyroid precursors originate from the pharyngeal region proximal to the *hand2*-expressing mesoderm cells (e.g., cardiac mesoderm), it is conceivable that *hand2* drives the expression of mesodermal factors with a permissive action in thyroid specification. Intriguingly, the ectopic overexpression of FGF rescues the thyroid defects of *hand2s6* mutants, suggesting that FGF acts in parallel or downstream the *hand2* signaling (93).

Furthermore, it has been reported that the overexpression of FGFs or FGF receptor strongly interfere with endoderm formation, whereas the inactivation of FGF signaling through the microinjection of antisense morpholinos is associated with an increased number of endodermal precursors. Interestingly, FGF inhibition is able to partially recover both endoderm induction and thyroid defects of *cas^ta56^* mutants, indicating that the proper endoderm formation in response to Nodal signaling is negative regulated by FGF (56).

In addition to those previously observed, Haerlingen and colleagues (9) described that the chemical inhibition of FGF during gastrula stages is associated with an enhanced expression of *nkx2.4b* in the thyroid primordium at 28 hpf, whereas the administration of the same compounds during somitogenesis completely abolish the specification of thyroid primordium, suggesting that these permissive factors act during critical developmental windows (9).

### Bone Morphogenic Proteins

The role of BMPs regulating mesoderm and ectoderm specification is well documented but only few studies explain its function during endoderm patterning (95). In mice, BMP signaling is required for definitive endoderm formation and migration of the anterior visceral endoderm, as evidenced by alterations of endoderm morphogenesis after depletion of *Bmp1a* (96, 97). Tiso and colleagues (2002) found that, in zebrafish, BMP contributes the dorsal-ventral patterning of the embryo. Interestingly, increased levels of BMP by *bmp2* mRNA microinjection have no consequences on the number of endoderm precursors but affect the expression of *her5*, a critical factor for antero-posterior patterning. A gradient of BMP signaling seems to define the extension of *her5* territory during gastrulation and thus, the allocation of anterior endodermal precursors (98). By contrast, Poulain et al. (56) described that the overexpression of a cocktail of *bmp2*, *bmp4* and *bmp7* strongly diminishes the number of endoderm precursors, whereas they are only slightly increased after the inhibition of BMP by the overexpression of *noggin* mRNA. Interestingly, the simultaneous inhibition of BMP and FGF signaling results in a massive increment of endodermal precursors (56). These studies give us new information about role for BMP in controlling endoderm induction and highlight that both positive (by Nodal) and negative (by BMP and FGF) signals are required for proper endoderm patterning.

Haerlingen et al. (9) described that the pharmacological inhibition of BMP during gastrulation severely affects thyroid specification, a phenotype that is difficult to explain given the posteriorizing BMP activity during endoderm formation (9). However, recent studies demonstrate that BMP is also important to induce ventralization of the foregut endoderm, and for that reason it might alters the specification of the thyroid bud that will later develop form that endodermal region (99). Intriguingly, these data suggest that the potential of the endodermal cells to became thyroid cells might be assigned already during gastrulation, and in this context, BMP activity seems to be required for such dorso-ventral patterning (9).

Moreover, similarly to FGF (see section 5.4), the chemical inhibition of BMP during somitogenesis impairs thyroid development by completely blocking the expression of early thyroid markers in the future thyroid region. Associated thyroid and cardiac defects are reported in embryos treated with antagonists of FGF (fgfr1) or BMP (alk1, 2, 3 and 6) receptors (9). Although in contrast to those observed in previous works that described the negative regulation of FGF and BMP signaling on thyroid specification, these results would add new pieces of this complex puzzle that will help the comprehension of the early molecular events driving thyroid development. It is plausible that the block FGF or BMP signaling during somitogenesis can perturb cardiac mesoderm development or its positioning relative to the endoderm, which in turn affects the induction of endodermal thyroid precursors as a consequence of an alteration in local signal activities.

Very recently, using the zebrafish reporter lines Tg(*dusp6:*d2EGFP) and Tg(*BRE:*dmKO2), Haerlingen et al. (16) mapped the activity of FGF and BMP in the foregut endoderm during early thyroid development (16). FGF signal is detectable around 18/19 hpf and co-localizes with the *pax2a^+^* thyroid precursors, whereas BMP co-localizes with *nkx2.4b* in the thyroid precursors at 22-24 hpf, near the heart tube. This suggests a possible correlation between FGF and *pax2a*, and BMP and *nkx2.4b* activation, and further supports the cardiac origins of FGF and BMP signals in regulating thyroid development (15, 56). To further dissect the dynamic activity of FGF and BMP, the authors also performed a 3-hours treatment with FGF or BMP inhibitors at early, middle, and late segmentation stages (16). In contrast to the strong reduction *nkx2.4b* and *tg* expression after FGF inhibition during the whole segmentation stage (9), the short-treatment with an FGF inhibitor do not significant alters the expression of TTFs.

However, the BMP inhibition of embryos at early and late segmentation stages leads to the absence of thyroid progenitors, similar to that observed after BMP inhibition during the entire segmentation stage. During the early and late segmentation periods the lack of BMP activity is associated with the absence or strong reduction of TTFs expression, whereas TTFs levels appear largely unaffected at the mid-segmentation period. These new findings indicate that FGF activity is required during the whole segmentation period, whereas BMP could play a biphasic role during zebrafish segmentation (16).

Interestingly, the overexpression of *fgfr1* between the middle and late stages of somitogenesis results in an increment of thyroid progenitors expressing *pax2a*, but not *nkx2.4b*. In parallel, the overexpression of *bmp2b* during early segmentation is associated to a corresponding increment of *nkx2.4b* ^+^ precursors and thyroid enlargement that abnormally elongates along the AP axis, likely due to major left-right asymmetry defects. Since *pax2a* is expressed earlier than *nkx2.4b* during thyroid specification, these data suggest that a coordinate activity of FGF and BMP governs early zebrafish thyroid development, where BMP signal during early segmentation acts upstream to FGF (16).

The importance of FGF and BMP signaling in thyroid fate decision has been also described in stem cell-based experiments by the Kotton group. Longmire et al. (90) established a protocol to efficiently isolate *Nkx2.1* ^+^ lung and thyroid lineages derived from induced pluripotent stem cells (iPSCs) in culture supplemented with FGF, BMP and Wnt (90). Kurmann and colleagues (2015) confirmed that the combinatorial effect of FGF and BMP is sufficient for *Nkx2.1^+^* endodermal-derived lineage differentiation with thyroid potential from embryonic stem cells (ESCs). Also, iPSCs can be fully reprogrammed into endodermal progenitors with thyroid potential by exposure to FGF2 and BMP4. Interestingly, the sorting of iPSCs expressing both *Nkx2.1* ^+^ and *Pax8^+^* give rise to thyroid epithelial cells expressing *Nkx2.1*, *Pax8*, *Foxe1*, *Hhex*, and *Tg*. Also, the treatment of iPSCs with BMP (Dorsomorphin) or FGF (LY) inhibitors reduces their capacity to express mature thyroid markers (89). Additional findings from Serra et al. (100) demonstrated that Wnt and BMP signaling are required to specify lung progenitors, whereas FGF and BMP signaling appear to be necessary to promote the specification of the thyroid lineage (100).

The ability to specifically isolate lung or thyroid competent lineages with ability to differentiate and reorganize into organ-specific functional units (bronchial tree/alveoli and thyroid follicles, respectively) makes a strong basis for further studies in developmental biology and regenerative medicine (100).

### Wnt Pathway

Wnt pathway is a highly conserved signal transduction pathway involved in critical events of embryogenesis, such as cell fate decision, cell differentiation, and body axis determination during embryonic development (101). The Wnt cascade integrates signals from other pathways, including FGF and BMP (102, 103). Wnt ligands are secreted glycoproteins that bind to the N-terminal extracellular domain of the Frizzled receptors, forming a large surface complex with the co-receptor LPR5/6. The Wnt receptor complex activates the canonical Wnt/ß-catenin pathway, or *via* the Wnt/Ca^2+^ or Wnt/planar cell polarity signaling pathways (104, 105). The functions of Wnt/ß-catenin pathway have been extensively studied in heart development, where it promotes precardiac mesoderm specification in cardiomyocyte precursors in zebrafish blastula. Intriguingly, Wnt inhibits cardiomyocyte differentiation at the gastrula stage, highlighting the importance of temporal activity of Wnt signaling during embryonic development (105). Regarding endoderm specification, it has been reported that in *Xenopus*, an antero-posterior gradient of Wnt activity seems to be necessary for anterior patterning of liver, lung, and thyroid, and posterior endoderm patterning of intestine (106). In zebrafish, a source of Wnt signal is required for proliferation of liver progenitors (107).

Very recently, the group of Costagliola reported that the overactivation of Wnt during early zebrafish development results in an impaired specification of both cardiac and thyroid tissues (9, 15). Embryos treated with BIO and AZA (Wnt up-regulator) at the thyroid anlage stage (28 hpf) show a reduction of *nkx2.4b* expression in the prospective thyroid region, in a dose-dependent manner. Embryos treated in later stage (55 hpf) exhibit a tiny thyroid primordium with few *tg*-positive cells. However, when the timing of drug treatment is shifted at early developmental stages, the thyroid defects primarily arise during gastrula and early somitogenesis. The block of cardiomyocyte differentiation by a specific morpholino (mef2c/d-MO) produces a similar thyroid phenotype, further supporting the view that altered signaling between cardiac mesoderm and endodermal thyroid precursors is associated with impaired thyroid specification. The thyroid defect is partially restored by BMP overactivation in these models, thus confirming that a source of BMP from cardiac mesoderm is critical for thyroid specification (15).

## Discussion

The huge effort of thyroid research in the last few decades successfully identified several genes and signaling pathways involved in intrinsic or extrinsic control of thyroid development. However, a very limited number of human CH cases with TH can be explained by mutations in these genes.

Targeted inactivation of genes involved in foregut development usually leads to growth arrest or premature death in mice (42). Thus, the availability of additional models is mandatory for study the early events that govern thyroid organogenesis.

As summarized in the present review, our knowledge of the earliest events regulating thyroid development, in particular from the initial regionalization and induction of the endoderm to thyroid anlage specification, has been moved forward by studies in zebrafish. What appeared clear from these findings is that the onset of the thyroid gland is more complex than initially expected. Several intrinsic and extrinsic signals appear to act in synergy to drive the cellular fate of the differentiating endoderm. Moreover, some signaling pathways (e.g., Notch and Sonic Hedgehog) seem to play roles during different phases of thyroid development: restricting the number of endodermal cells committed to thyroid fate and, subsequently, controlling differentiation and migration of thyroid follicles. Notably, such direct effects on thyroid specification seems to occur when Notch receptors bind the *jag1* ligands (expressed in thyroid tissue) or Shh signal cascade culminates in the activation of *glis3* (expressed in pharyngeal endoderm) (77, 83).

Several recent works have demonstrated the crucial role of surrounding tissues (e.g., lateral plate mesoderm) in the control of the initial steps of endoderm formation, as well as in the acquisition of thyroid competence of endodermal progenitors (1, 2, 9, 15, 18, 33). Noteworthy, the detailed mapping of spatial and temporal expression of TTFs and FGF/BMP described by Costagliola group has significantly increased our comprehension about the fine regulation and crosstalk between mesoderm and endoderm. Of particular interest, the colocalization of FGF and *pax2a* signals in cells located in the foregut endoderm at 18-20 hpf, would help the identification of endodermal precursors with a thyroid competence before the onset of the thyroid primordium, thus allowing a more targeted study of thyroid precursors (16).

However, one of the major problems hampering a complete understanding of the specific contribution of each signaling pathway during the early events of thyroid development are the presence of discordant phenotypes observed in numerous zebrafish models generated by different experimental protocols. For instance, Poulain et al. (56) describe that the inhibition of FGF signaling by microinjection of morpholinos results to an increase of endodermal precursors, whereas Haerlingen et al. (9) show that the chemical inhibition of FGF during somitogenesis completely abolish the specification of thyroid primordium (9, 56). Although it is difficult to compare models produced with different methodology, the analysis of their similarities and differences would be informative in terms of gene dosage, spatial and temporal activity, as well as maternal and zygotic contribution of a gene of interest. Nevertheless, different thyroid phenotypes are described after the treatment of zebrafish embryos with the same inhibitor, as in the case of DAPT (9, 77) or cyclopamine (9, 84). Indeed, an additional effort should be made to optimize the experimental protocols and understand possible unknown off-target effects of such inhibitors. In **Table 1**, we summarize all of the available zebrafish models generated for studying endoderm induction and thyroid specification.

**Table 1:** Morphogenetic pathways involved in endoderm induction and specification of thyroid primordium.

Signaling Pathwav	Gene	Experimental condition	Endoderm and/or Thyroid phenotypes	References
Nodal	*oep*	Mutant fish line (*oep^tz57^*)	Failure of mesendodermal cells commitment, loss of endoderm specification, absence of thyroid primordium	(59, 60)
	*cyc*	Mutant fish line (*cyc^m294^*)	Incomplete development of pharyngeal endoderm, reduced number of functional thyroid follicles	(59, 61)
	*cyc, hhex*	Mutant fish line (*cyc^mb16^*)	Absence specification of thyroid primordium, no functional thyroid follicles	(59, 63)
	*sox32*	Mutant fish line (*cas^ta56^*)	Lack of endodermal precursors	(59, 63)
	*bon*	Mutant fish line (*bon^s9^*)	Reduced number of endodermal precursors, absence of gut tube	(59, 63)
	*gata5*	Mutant fish line (*fau^s26^*)	Reduced number of endodermal precursors	(57, 59)
	*tgf-β*	LY364947 and SB505124 inhibitors	Alteration of anterior endoderm, slight reduction of thyroid progenitors	(9)
Notch	*notch-NICD*	Conditional KI fish line [late somitogenesis]	Reduced thyroid primordium specification, low number of functional follicles	(77)
	*mib*	Mutant fish line (*Mib^ta52b^*)	Increased thyroid primordium specification, increased number of functional follicles	(77)
	*jagla, jalb*	Morphilino-mediated KD, Mutant fish line (jag1b*^b1005^*)	*Jagla*: reduced proliferation of differentiated follicles; *jaglb*: reduced thyroid promordium specification	(77)
	*γ-secretase*	DAPT inhibitor [gastrula]	Increased thyroid primordium specification, increase number of functional follicles	(77)
	*γ-secretase*	LY411575, RO4929097 inhibitors [gastrula, somitogenesis]	No significant abnormality detected	(9)
Sonic Hedgehog	*shh*	Morpholino-mediated KD	Abnormal thyroid migration	(8)
	*glis3*	Morpholino-mediated KD	Reduced thyroid primordium specification, low number of functional follicles	(83)
	*ptch1*	Cyclopamine inhibitor [gastrula]	Reduced thyroid primordium specification	(83)
	*ptch1*	Cyclopamine inhibitor [gastrula, somitogenesis]	Slight impairment of follicle migration	(9)
	*smo*	Purmophamine, SAG inhibitors [gastrula, somitogenesis]	No significant abnormality deteted	(9)
FGF	*fgf8*	Morpholino-mediated KD, Mutant fish line (*ace^ti282a^*)	Increased number of endodermal precursors, reduced expression of TTFs, loss of functional follicles	(56, 93)
	*hand2*	Mutant fish lines (han^s6^, han^c99^)	Reduced or absent expression of thyroid markers, loss of fuctional follicles	(93, 94)
	*fgfr 1*	SU5402, PD166866 inhibitor [gastrula, somitogenesis]	Gastrula: increased thyroid primordium specification; somitogenesis: no thyroid primordium specification	(9)
	*fgfr 1*	PD166866 inhibitor [early, middle, and late somitogenesis]	No significant changes detected in TTFs expression	(16)
	*fgfr 1*	Conditonal KI fish line [early, middle, and late somitogenesis]	Middle/late: increment of thyroid progenitors that express *pax2a*	(16)
BMP	*bmp2b*	mRNA injection	No consequence of number of endoderm precursors	(98)
	*bmp2b, bmp4, bmp7*	mRNA injection	Reduction of endodermal precursors number	(56)
	*noggin*	mRNA injection	Slight increase of endodermal precursors number	(56)
	*alk2, and alk1-3 and 6*	DMH1, LDN193189 inhibitors [gastrula, somitogenesis]	Gastrula: altered specification of thyroid bud; somitogenesis: no thyroid primordium specification	(9)
	*alk2*	DMH1 inhibitor [early, middle, and late somitogenesis]	Early: absence of thyroid progenitors; middle: mild reduction of TFFs expression; late dramatic reduction of TFFs in thyroid primordium:	(16)
	*bmp2b*	Conditional KI fish line [early, middle, and late somitogenesis]	Early: increment of *nkx2.4b^+^* precursors and thyroid enlargement that abnormally elongate along the AP axis; middle/late: enlargement of the *pax2a^+^* thyroid precursor population and premature expression of *nkx2.4b*	(16)
Wnt	*gsk3a and gsk3β*	BIO and AZA activators [gastrula, somitogenesis, thyroid anlage, folliculogenesis]	Gastrula/somitogenesis: severe alteration of endoderm specification; thyroid anlage/folliculogenesis: reduction of thyroid primordium	(9, 15)
FGF + BMP	*fgf8, fgf17b, fgf24, and nogging*	Morpholino-mediated KD (for FGF) and mRNA injection (for BMP)	Massive increment of endodermal precursors population	(56)

For each signaling pathway, the gene (in italics), the experimental condition used to generate the zebrafish model, the associated endoderm and/or thyroid phenotype, and the corresponding references are reported. When available, the developmental stage selected to start the treatment with inhibitors are also reported in the squared parenthesis. AP axis, antero-posterior axis; KD, knockdown; KI, knockin; NICD, Notch Intracellular domain; TTFs, thyroid transcription factors.

Altogether, this work demonstrates that zebrafish is a valuable model for the investigation of the complex crosstalk between the several, and possibly redundant, factors that act through the expression of specific endodermal or mesodermal genes that guide the proper thyroid specification.

In conclusion, the described zebrafish models open new perspectives in understanding the early stages of thyroid development. These data represent a practical basis to set protocols for the generation of functional thyroid follicles from stem cells and will hopefully translate in an improved understanding of the pathogenesis of thyroid dysgenesis, the most frequent congenital endocrine defect and preventable cause of intellectual disability.

## Author Contributions

FM and GR performed literature search and drafted the manuscript and figures. LP revised and finalized the manuscript for submission. All authors contributed to the article and approved the submitted version.

## Funding

This work was partially supported by Ricerca Corrente funds of Istituto Auxologico Italiano, IRCCS (project Zebratir, # 05C102_2011).

## Conflict of Interest

The authors declare that the research was conducted in the absence of any commercial or financial relationships that could be construed as a potential conflict of interest.
